# Combining radiomic phenotypes of non-small cell lung cancer with liquid biopsy data may improve prediction of response to *EGFR* inhibitors

**DOI:** 10.1038/s41598-021-88239-y

**Published:** 2021-05-11

**Authors:** Bardia Yousefi, Michael J. LaRiviere, Eric A. Cohen, Thomas H. Buckingham, Stephanie S. Yee, Taylor A. Black, Austin L. Chien, Peter Noël, Wei-Ting Hwang, Sharyn I. Katz, Charu Aggarwal, Jeffrey C. Thompson, Erica L. Carpenter, Despina Kontos

**Affiliations:** 1 Department of Radiology, University of Pennsylvania, Philadelphia, PA 19104 USA; 2 Department of Radiation Oncology, University of Pennsylvania, Philadelphia, PA 19104 USA; 3 Division of Hematology and Oncology, Department of Medicine, University of Pennsylvania, Philadelphia, PA 19104 USA; 4 Department of Biostatistics, Epidemiology and Informatics, University of Pennsylvania, Philadelphia, PA 19104 USA; 5 Section of Interventional Pulmonology, Department of Medicine, University of Pennsylvania, Philadelphia, PA 19104 USA; 6 Computational Biomarker Imaging Group (CBIG), Department of Radiology, University of Pennsylvania, Rm D702 Richards Bldg., 3700 Hamilton Walk, Philadelphia, PA 19104 USA

**Keywords:** Non-small-cell lung cancer, Prognostic markers

## Abstract

Among non-small cell lung cancer (NSCLC) patients with therapeutically targetable tumor mutations in epidermal growth factor receptor (*EGFR*), not all patients respond to targeted therapy. Combining circulating-tumor DNA (ctDNA), clinical variables, and radiomic phenotypes may improve prediction of EGFR-targeted therapy outcomes for NSCLC. This single-center retrospective study included 40 *EGFR*-mutant advanced NSCLC patients treated with EGFR-targeted therapy. ctDNA data included number of mutations and detection of *EGFR* T790M. Clinical data included age, smoking status, and ECOG performance status. Baseline chest CT scans were analyzed to extract 429 radiomic features from each primary tumor. Unsupervised hierarchical clustering was used to group tumors into phenotypes. Kaplan–Meier (K–M) curves and Cox proportional hazards regression were modeled for progression-free survival (PFS) and overall survival (OS). Likelihood ratio test (LRT) was used to compare fit between models. Among 40 patients (73% women, median age 62 years), consensus clustering identified two radiomic phenotypes. For PFS, the model combining radiomic phenotypes with ctDNA and clinical variables had c-statistic of 0.77 and a better fit (LRT *p* = 0.01) than the model with clinical and ctDNA variables alone with a c-statistic of 0.73. For OS, adding radiomic phenotypes resulted in c-statistic of 0.83 versus 0.80 when using clinical and ctDNA variables (LRT *p* = 0.08). Both models showed separation of K–M curves dichotomized by median prognostic score (*p* < 0.005). Combining radiomic phenotypes, ctDNA, and clinical variables may enhance precision oncology approaches to managing advanced non-small cell lung cancer with *EGFR* mutations.

## Introduction

The discovery of activating mutations and the development of targeted therapies has improved survival in patients with non-small cell lung cancer (NSCLC)^1^. Mutation detection by tissue and circulating tumor DNA (ctDNA) next-generation sequencing (NGS) guides therapy selection both at initial diagnosis and disease progression. Epidermal growth factor receptor (*EGFR*) mutations are the most common therapeutically targetable variants in NSCLC, and treatment with an *EGFR* tyrosine kinase inhibitor (TKI) has shown superior efficacy compared to standard chemotherapy in mutation-positive patients^2^. However, primary resistance occurs in 20–30% of patients^3^. Ultimately, all patients develop acquired resistance to EGFR-directed therapies and an active area of research is the use of novel combination therapies, including antibodies against c-met, poly-adenosine diphosphate ribose polymerase inhibitors and antiangiogenic therapies along with EGFR-TKIs to improve long-term efficacy^4,5^.

Tumor heterogeneity is thought to play a role in TKI response and is associated with poor outcome^6–9^, as *EGFR* mutations may be suboptimal targets when they co-occur with genetic alternations or are subclonally expressed^8,9^. Small tissue biopsies may not fully reflect tumor heterogeneity and can often be difficult to obtain^10,11^, with tissue NGS only able to be completed for as few as 50% of patients^12^. Thus, developing non-invasive tests to assess the likelihood of response to an EGFR-TKI is critical for therapy selection. Studies have shown that ctDNA analysis represents a non-invasive biomarker that can improve targetable mutation detection, and that ctDNA molecular heterogeneity predicts clinical outcome^13–15^. Although useful clinically, however, ctDNA sensitivity remains less than ideal^13^.

An emerging non-invasive approach to characterize tumor heterogeneity is to analyze tumor imaging phenotypes^16,17^. Radiomics analysis enables the detection of tumor imaging features and patterns of intra-tumor heterogeneity not appreciable by the human eye, increasing the wealth of information from radiological imaging. Studies specifically suggest that radiomic analysis may provide novel prognostic markers related to gene-expression patterns and responder signatures for NSCLC patients receiving targeted therapy^18–31^. Most studies to date have focused on using radiomic analysis on computed tomography (CT) and/or positron emission tomography (PET)/CT data to predict *EGFR* mutation status, using statistical modeling or machine learning approaches for reducing the high dimensionality of radiomic features^19,21–29,32^. More recently deep learning approaches have also been used to predict outcomes after TKI therapy for NSCLC^31,33^. While this field is rapidly developing, a question still remains as to which extent radiomic analysis can complement established prognostic markers for TKIs, as most studies have either evaluated radiomic features in the absence of established prognostic biomarkers or have only examined surrogate endpoints, such as *EGFR* mutation status, rather than actual patient outcomes. In addition, and to the best of our knowledge, no studies have evaluated radiomic analysis in the context of complementing liquid biopsy-based assessment, which is another promising non-invasive tool for characterizing tumor heterogeneity when predicting EGFR-TKIs response.

The purpose of our study was to determine the feasibility of integrating radiomics features with ctDNA next-generation sequencing data to predict TKI outcomes in *EGFR* mutant NSCLC. Our approach combines unsupervised hierarchical clustering and principal component analysis (PCA) of radiomic features extracted from clinically acquired CT scans, to arrive at two distinct radiomic phenotypes. Our hypothesis is that integrating these radiomic phenotypes with ctDNA and clinical variables can improve assessment of tumor heterogeneity and outcome prediction to *EGFR*-targeted therapy for metastatic NSCLC.

## Materials and methods

### Study sample and data

**Figure 1:**
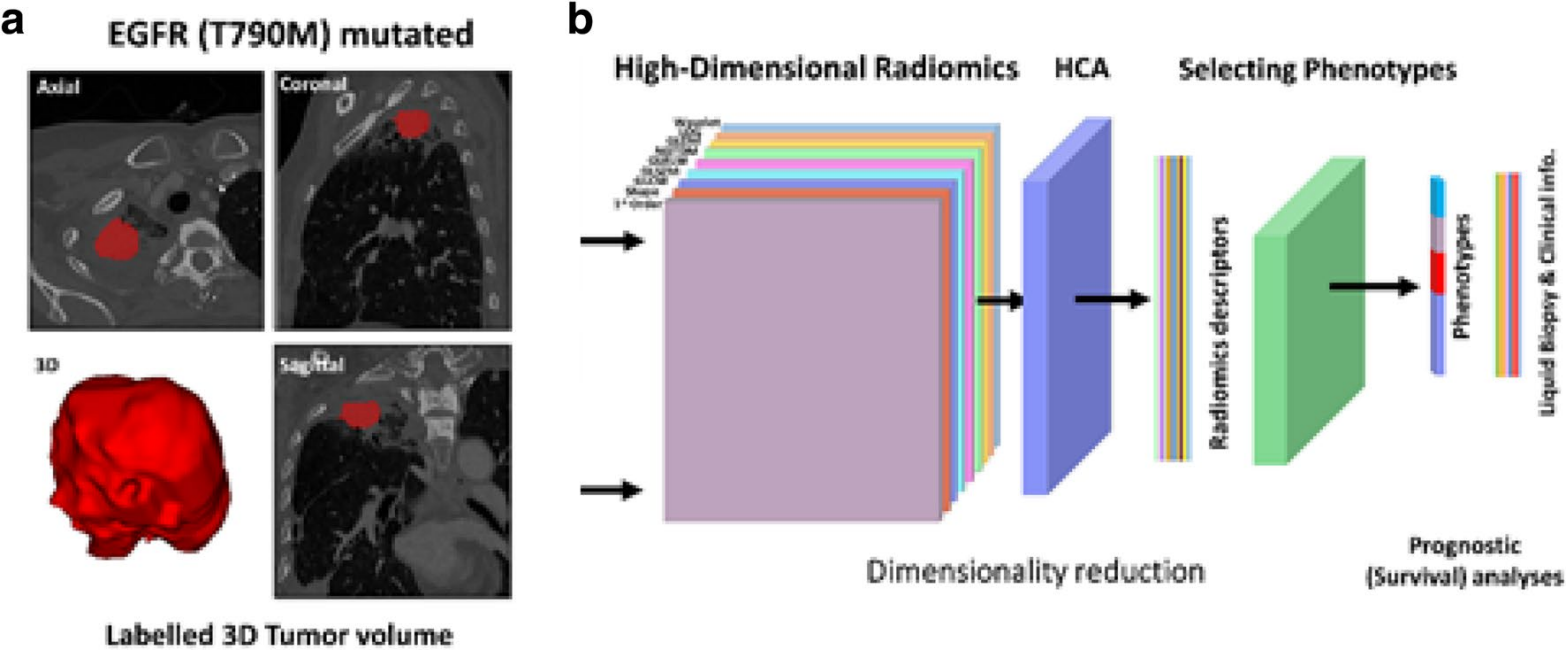
Tumor segmentation and radiomic analysis. (**a**) Example of segmentation of a tumor expressing the epidermal growth factor receptor (EGFR) T790M mutation. (**b**) Workflow of radiomics analysis where the tumor is segmented in 3D, followed by radiomic feature extraction, and two-level hierarchical clustering to first reduce feature dimensionality and then cluster the derived radiomic signatures into distinct tumor phenotypes.

This single-center, retrospective, observational study was conducted at the University of Pennsylvania from October 2016 to February 2019 and was approved by the Institutional Review Board with Health Insurance Portability and Accountability Act waiver of informed consent. All methods in this study were in accordance with the Declaration of Helsinki and informed consent was obtained from all the participants. Patients with metastatic NSCLC that had an actionable *EGFR* mutation detected by ctDNA next-generation sequencing and also had CT imaging data available for radiomic analysis were included. Based on these criteria, a total of 40 *EGFR*-mutant advanced NSCLC patients were included in the study. All patients were treated with the EGFR-TKI indicated by the clinical ctDNA next-generation sequencing result either at the time of diagnosis (n = 23) or suspected progression on a front-line EGFR-TKI (n = 17). The patients starting an EGFR-TKI at the time of diagnosis received afatinib (n = 8), erlotinib (n = 5), gefitinib (n = 1), or osimertinib (n = 9). All patients who had experienced progression on a front-line EGFR-TKI received osimertinib (n = 17). Baseline demographics, clinical data, including ctDNA targeted next-generation sequencing results (Guardant360 73 gene panel), and baseline CT scans were collected from the electronic medical record. ctDNA features measured included: allele fraction of the therapeutically targetable driver mutation, total number of co-existing mutations detected, and whether the *EGFR* T790M mutation was detected. Chest CT data included a total of 7 contrast-enhanced and 33 non-contrast enhanced scans, of which 24 were acquired with Siemens and 16 with a General Electric scanner (Supplementary Table S1). A board-certified, fellowship-trained thoracic radiologist (S.I.K.) with 18 years of clinical experience manually segmented the tumor area using the semi-automated ITK-SNAP software (version 3.6.0) (Fig. 1a)^34^.

### Radiomic feature extraction

A total of 429 radiomic features were extracted from each tumor’s entire volume using the PyRadiomics library^35^, representing nine type of descriptors: (1) First-order statistics, capturing the voxel grey-level intensities within a neighborhood. (2) Shape-based descriptors of the three-dimensional size and shape of the tumor measured on the whole tumor volume. (3) Gray level co-occurrence matrix features, calculated based on second-order joint probability functions of voxel intensities in a particular spatial relation, for all intensities and many spatial relations. (4) Gray level size zone matrix features, similar to gray level co-occurrence matrix features but rotation-independent. (5) Gray level run length matrix features, based on quantifying gray level runs as the lengths of consecutive pixels. (6) Gray level dependence matrix features, calculated as the number of connected voxels within a specified distance. (7) Neighboring gray tone difference matrix features, rotation-independent features based on gray-level relationships between neighboring voxels (for a certain distance between voxels). (8) Laplacian of Gaussian features, capturing information about edge detection in a smoothed image. (9) Wavelet features, giving information on the location, direction, and frequency of gray-level changes. All features were z-scored prior to further analysis.

### Radiomic phenotype identification

We used the extracted features as input to a two-level hierarchical clustering algorithm: first, features were clustered and principal component analysis was used to reduce dimensionality and construct a feature-vector signature reflecting each tumor’s imaging phenotype (i.e., feature-level clustering); then the derived feature vector signatures were clustered (i.e., tumor-level clustering) to identify intrinsic tumor phenotypes (Fig. 1b). Specifically, for Pearson’s correlation $r$ between any two features, we defined $1-{r}^{2}$ as a metric for the distance between the z-scored radiomic features, with strongly covarying features being closer. Using this metric, we performed unsupervised hierarchical clustering, applying the maximum distance linkage on the extracted features^36^. To determine the optimal number of feature clusters we used consensus clustering^37^ with a 10% cutoff for minimum change in the cumulative density function. We then performed PCA on each identified feature cluster and retained the first principal component (PC) from each cluster for all subsequent statistical modeling. As the features in each cluster covary strongly, the first PC should capture the dominant information in each feature cluster. Where $k$ is the number of feature clusters, dimensionality is thus reduced from 429 total radiomic features measured to $k$, with $k$ substantially lower than 429. Using the same unsupervised hierarchical approach as described above^36,37^ we used these derived PC feature signatures to cluster our sample into distinct radiomic tumor phenotypes, where the optimal number of phenotype clusters was deemed by consensus clustering^37^.

### Statistical analysis

We used Kaplan–Meier (K–M) curves and log-rank test to assess the univariable association between radiomic phenotype and each of progression-free survival (PFS) and overall survival (OS). We also used K–M curves to assess the association between these outcomes and each of the established prognostic clinical covariates of age, smoking status, and Eastern Cooperative Oncology Group (ECOG) performance score; patient line of therapy (first versus second or third); and the ctDNA-derived number of mutations. Further, Cox proportional-hazards regression models provided hazard ratios (HRs) and *p* values for the effect of each of these covariates. Retaining number of mutations and all other covariates that gave *p* ≤ 0.2 for association in a univariable model, we examined multivariable models both with and without radiomic phenotype. We evaluated Cox models using the likelihood-ratio test (LRT) both versus the null model, and, for the multivariable model, versus the nested model without radiomic phenotype. Finally, model discrimination capacity was assessed via the concordance statistic (c-statistic), as modified by Uno et al.^38^, with a time horizon for each event type of τ = the longest time-to-event for that event type. As a subsidiary analysis, we also examined the K-M curves for PFS and OS versus what line of therapy a patient received—first versus second or third—and for radiomic phenotype within strata of line of therapy.

To evaluate possible confounding, variations in CT acquisition including contrast-enhanced versus non-contrast-enhanced imaging, helical pitch, X-ray voltage, and tube current (Supplementary Table S1) were also tested for association both with radiomic phenotype via Fisher’s exact and Mann–Whitney–Wilcoxon tests and with outcome via K–M curves.

Statistical significance was tested throughout all analyses versus *α* = 0.05. We performed all data manipulation, statistical analysis, and plotting using Python (Ver. 3.7, Anaconda) and the R programming language (Ver. 3.5.1)^39–41^.

## Results

### Study sample

The median age in our study sample was 62 years, with 29 (72.5%) women, 21 former smokers (52.5%) and 19 never smokers (47.5%). All patients had a therapeutically targetable *EGFR* mutation detected by clinical ctDNA testing, including: *EGFR* exon 19 deletion, *EGFR* L858R, *EGFR* G719C/S768I, *EGFR* Exon 20 insertion, and *EGFR* T790M. Patients were followed for a median time of 328 days, range 29–835. All patients received the *EGFR* inhibitor indicated by their ctDNA testing, with 23 (57.5%) receiving the drug in the front-line setting and 17 (42.5%) in the later line setting (Table 1). Of the 40 patients, 11 died and 29 were censored (maximum time to death 676 days, median 339); 20 showed disease progression and 20 were censored (maximum time to progression 511 days, median 231). There was no statistically significant difference for any of the clinical covariates between phenotypes except for first versus later (second or third) line of TKI therapy (*p* = 0.01) (Table 1). The majority (15 of 23) of patients receiving front-line therapy were classified into phenotype 2, and the majority (13 of 17) of patients receiving a later line therapy into phenotype 1.

**Table 1:** Patient characteristics.

Characteristic	Radiomic phenotype 1 *n* = 21 of 40 (52.5%)	Radiomic phenotype 2 *n* = 19 of 40 (47.5%)	Overall/total *N* = 40	*p*
Age, median (IQR) (range)	64 (58–69) (46–82)	59 (56–68) (45–80)	62 (56–69) (45–82)	0.3^1^
**Smoking status**				0.22^2^
Former	9 (42.9%)	12 (63.2%)	21 (52.5%)	
Never	12 (57.1%)	7 (36.8%)	19 (47.5%)	
**Sex**				0.73^2^
F	16 (76.2%)	13 (68.4%)	29 (72.5%)	
M	5 (23.8%)	6 (31.6%)	11 (27.5%)	
ECOG performance, mean (median) (range)	0.57 (0.0) (0–2)	0.53 (1.0) (0–1)	0.55 (0.5) (0–2)	1^1^
**Line of therapy**				0.01^2^
First	8 (38.1%)	15 (78.9%)	23 (57.5%)	
Second or third	13 (61.9%)	4 (21.1%)	17 (42.5%)	
**EGFR mutation status**				0.07^2^
Exon 19 deletions	5 (23.8%)	9 (47.4%)	14 (35.0%)	
Exon 20 insertion	1 (4.8%)	0 (0.0%)	1 (2.5%)	
G719C/S768I	0 (0.0%)	1 (5.3%)	1 (2.5%)	
L858R	3 (14.3%)	5 (26.3%)	8 (20.0%)	
T790M	12 (57.1%)	4 (21.1%)	16 (40.0%)	
Number of mutations, mean (sd) (range)	4.1 (1.76) (2–8)	3.9 (1.81) (1–8)	4.0 (1.76) (1–8)	0.8^3^
EGFR mutation allelic fraction, mean (range)	6.8 (0–32)	8.4 (0–53)	7.6 (0–53)	0.17^1^

^1^p value by Mann–Whitney–Wilcoxon test, two-sided.

^2^p value by Fisher’s exact test, two-sided.

^3^p value by Welch’s t-test, two-sided.

### Radiomic phenotype identification

**Figure 2:**
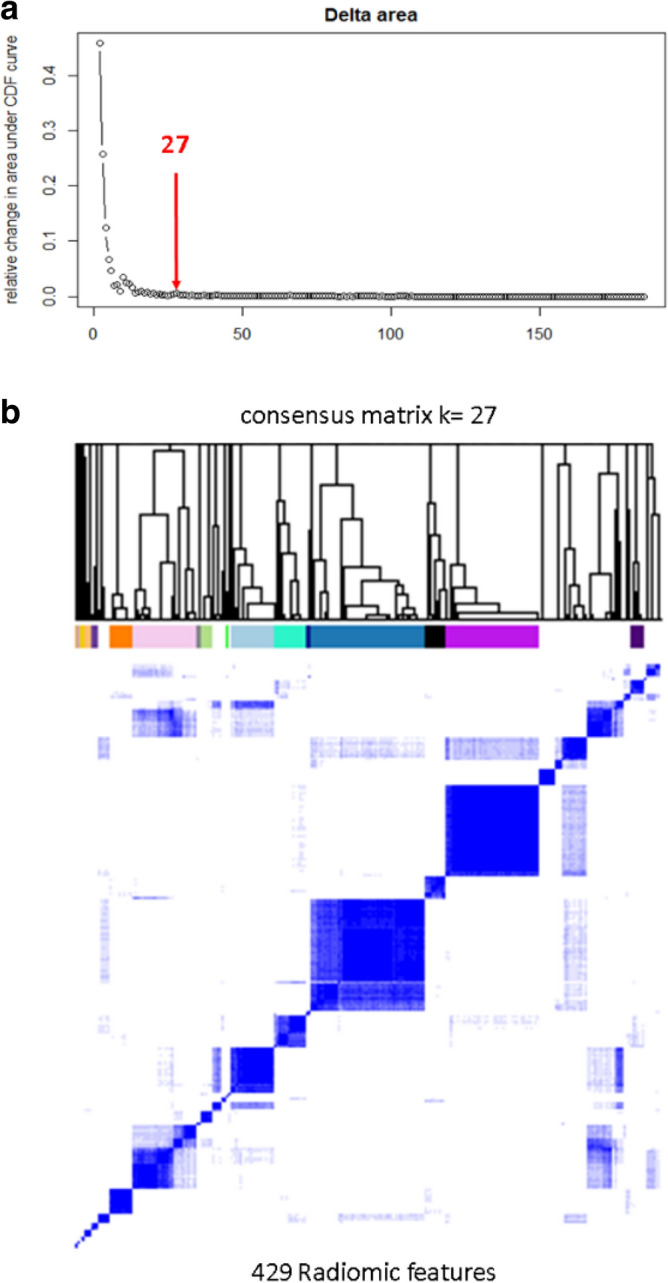
Selection of derived radiomic features. Cumulative distribution function (CDF) and consensus clustering are used to determine the optimum number of clusters of radiomic features. The red arrow in (**a**) represents the point (*k* = 27) where the relative change in CDF drops below 10%; (**b**) shows the clustered dendrogram corresponding to the 27 derived features.

**Figure 3:**
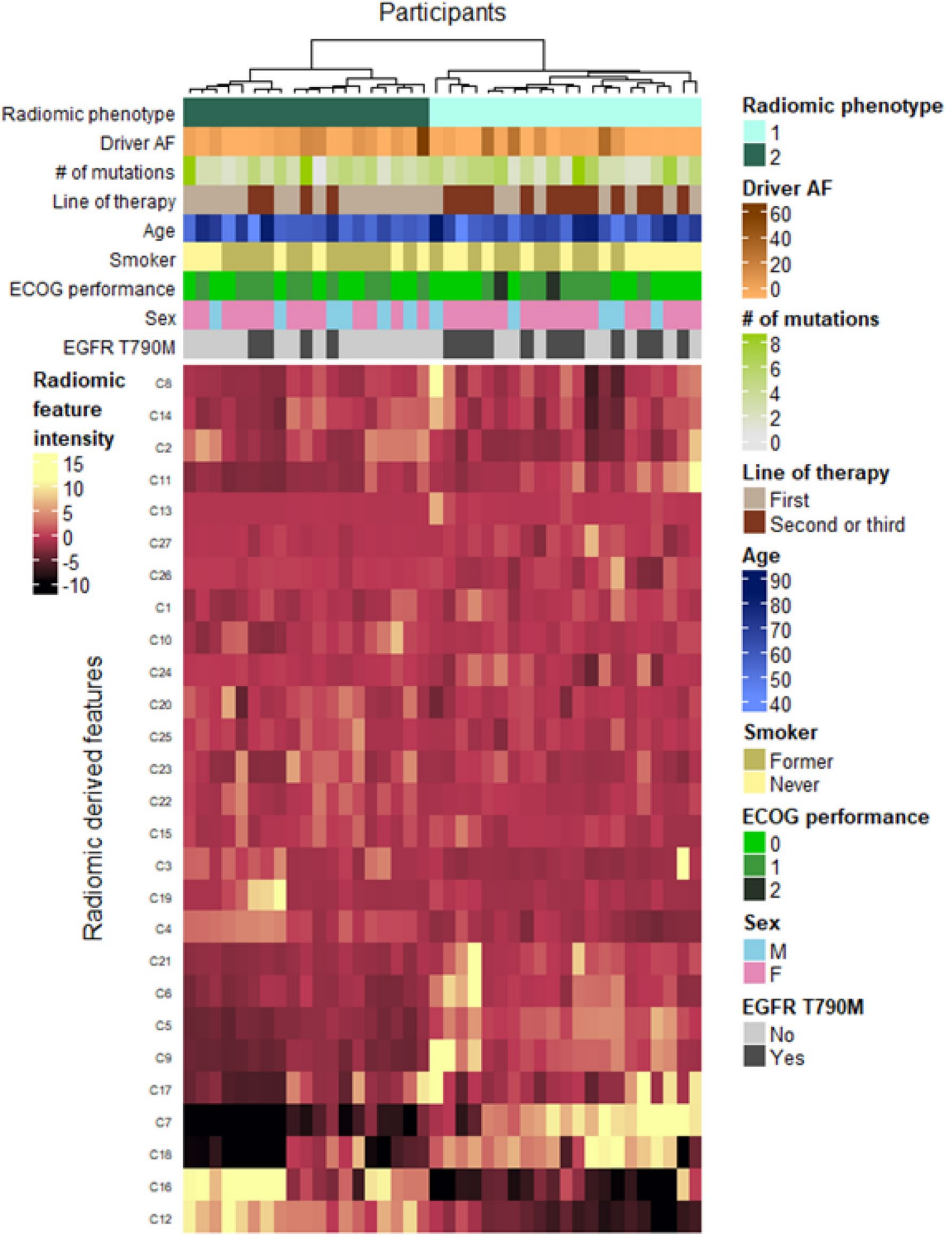
Heatmap of radiomic derived features. Unsupervised hierarchical clustering identifies two distinct, and statistically significant (p < 0.05), tumor radiomic phenotypes. Association of these phenotypes with study covariates is shown by the top colorbars. Driver AF is the percent allele fraction for the detected epidermal growth factor receptor (*EGFR*) driver mutation. *EGFR* T790M refers to those patients for whom the mutation was detected in circulating-tumor DNA.

From the 429 initially extracted radiomic features assessed, feature-level clustering with PCA gave *k* = 27 derived features (Fig. 2), when the relative change in area under the cumulative distribution function (CDF) fell below 10%. Subsequent tumor-level clustering identified two distinct radiomic phenotypes, with 21 tumors in phenotype 1 and 19 in phenotype 2 (Fig. 3) (*p* < 0.001 for SigClust test of two clusters versus one). No significant associations were found between CT acquisition parameters (including contrast-enhanced versus non-contrast-enhanced imaging) and phenotype or outcome (Supplementary Tables S2, S3, Supplementary Fig. S1).

### Radiomic phenotype association with outcomes

**Figure 4:**
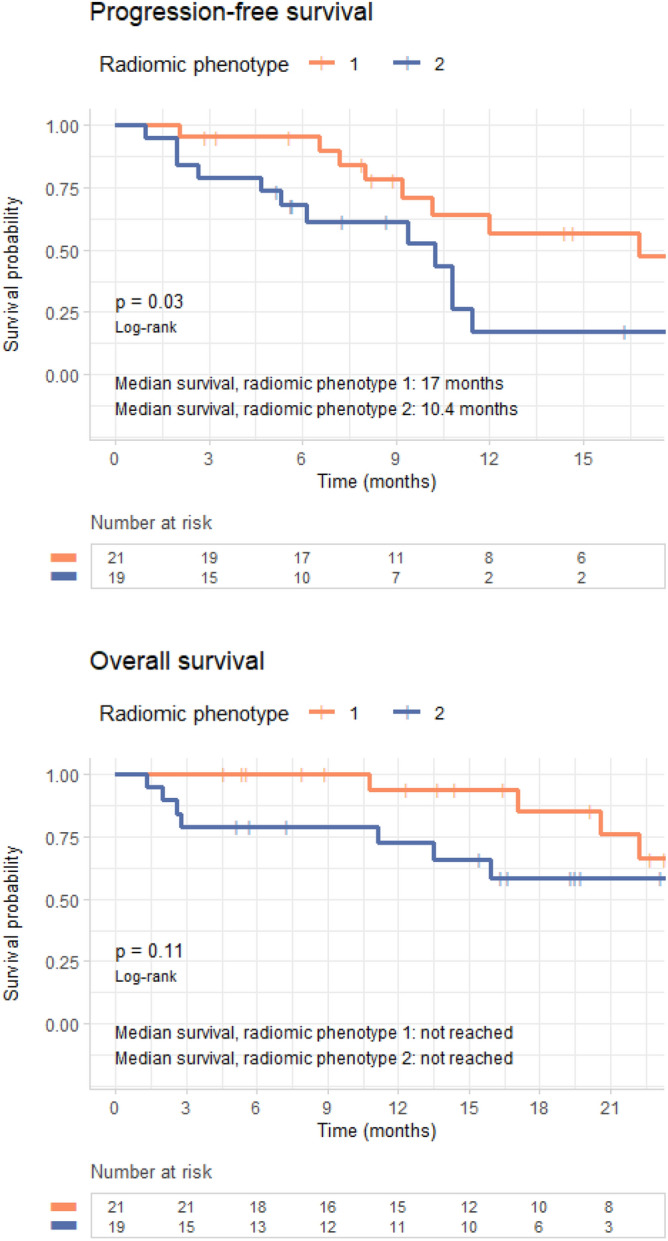
Survival analysis by radiomic phenotype. Progression-free survival (top) and overall survival (bottom) analysis for radiomic phenotypes.

**Figure 5:**
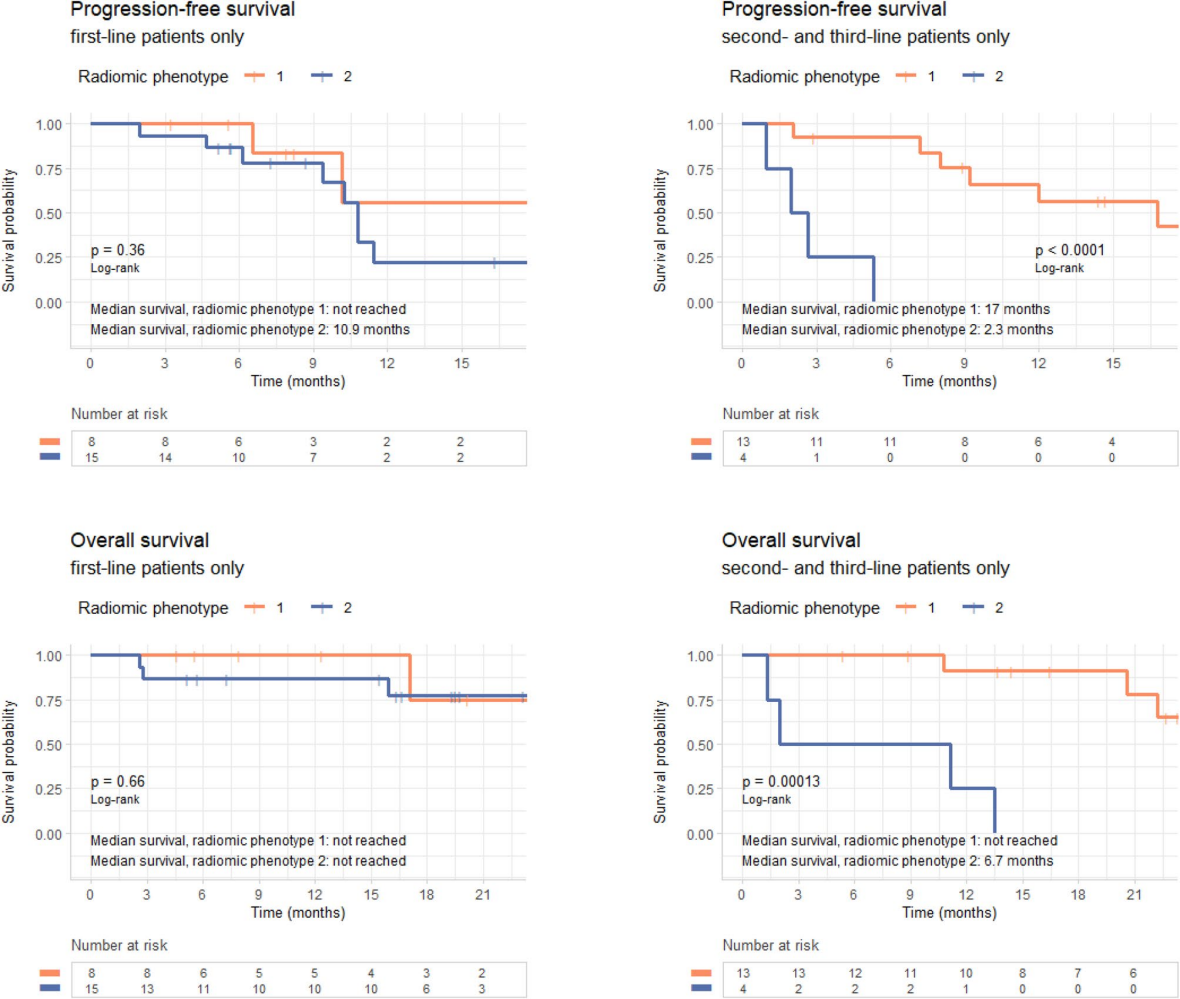
Survival analysis by line of therapy. Kaplan–Meier curves for (top row) progression-free survival and (bottom row) overall survival in first-line patients (left) and second- and third-line patients (right), showing that the radiomic tumor phenotypes can further sub-stratify patients in the second or third line of treatment.

Median PFS was 17 months for patients with radiomic phenotype 1 versus 10.4 months for those with phenotype 2 (median OS was not reached for either phenotype). The split between K–M curves for PFS resulted into log-rank *p* = 0.03; in a univariable Cox model, the HR 2.7 (95% confidence interval (CI) 1.1, 6.6) (*p* = 0.04) for tumors with radiomic phenotype 2 versus 1 (Fig. 4, Table 2). In OS, K–M curves dichotomized by phenotype resulted in a log-rank *p* = 0.11; in the corresponding univariable Cox model, the HR 2.7 (95% CI 0.8, 9.2) (*p* = 0.12) for tumors with phenotype 2 versus 1 (Fig. 4, Table 3). When PFS and OS were analyzed by line of therapy, radiomic phenotype showed statistically significant separation of the K–M curves for both outcomes in patients who received second or third line of therapy (*p* < 0.005), whereas there was no appreciable separation for patients who received front-line *EGFR*-targeted therapy (*p* = 0.36 and *p* = 0.66 for PFS and OS, respectively) (Fig. 5). The ECOG performance score also showed association with PFS and OS (PFS: HR 3.56 [95% CI (1.64, 7.73)], *p* < 0.005; OS: HR 2.91 [95% CI (1.17, 7.24)], *p* = 0.02). Smoking status showed *p* < 0.2 in univariable modeling and so, along with ECOG performance, was retained in the multivariable model (Tables 2, 3).

**Table 2:** Progression-free survival Cox regression hazard ratios.

Model	Covariate	Hazard ratio (95% CI)	*p*
Number of mutations	Number of mutations	0.99 (0.77, 1.28)	0.97
Smoking status	Smoking status (never versus former)	0.46 (0.18, 1.15)	0.1
ECOG performance score	ECOG performance status (per one increment in grade)	3.56 (1.64, 7.73)	< 0.005
Radiomic phenotype	Radiomic phenotype (2 versus 1)	2.66 (1.07, 6.64)	0.04
All covariates except radiomic phenotype	Number of mutations	0.86 (0.65, 1.15)	0.32
	Smoking status (never versus former)	0.47 (0.17, 1.31)	0.15
	ECOG performance status (per one increment in grade)	3.47 (1.56, 7.72)	< 0.005
All covariates	Radiomic phenotype (2 versus 1)	3.8 (1.35, 10.69)	0.01
	Number of mutations	0.91 (0.67, 1.23)	0.53
	Smoking status (never versus former)	0.75 (0.26, 2.14)	0.59
	ECOG performance status (per one increment in grade)	5.14 (1.99, 13.3)	< 0.005

**Table 3:** Overall survival Cox regression hazard ratios.

Model	Covariate	Hazard ratio	*p*
Number of mutations	Number of mutations	0.88 (0.61, 1.26)	0.47
Smoking status	Smoking status (never versus former)	0.19 (0.04, 0.89)	0.03
ECOG performance score	ECOG performance status (per one increment in grade)	2.91 (1.17, 7.24)	0.02
Radiomic phenotype	Radiomic phenotype (2 versus 1)	2.66 (0.76, 9.22)	0.12
All covariates except radiomic phenotype	Number of mutations	0.68 (0.44, 1.05)	0.08
	Smoking status (never versus former)	0.17 (0.03, 0.94)	0.04
	ECOG performance status (per one increment in grade)	2.87 (0.99, 8.3)	0.05
All covariates	Radiomic phenotype (2 versus 1)	3.88 (0.73, 20.5)	0.11
	Number of mutations	0.71 (0.45, 1.14)	0.16
	Smoking status (never versus former)	0.29 (0.05, 1.73)	0.17
	ECOG performance status (per one increment in grade)	4.43 (1.19, 16.55)	0.03

### Radiomic phenotype association with outcomes when combined with clinical and liquid biopsy data

Age, smoking status, and ECOG performance status are established prognostic factors for metastatic NSCLC^42,43^ that are considered clinically in selecting a patient’s therapy. While ctDNA NGS is often used to detect therapeutically targetable mutations, the association of other ctDNA measures, such as the number of mutations detected which may be a surrogate of tumor heterogeneity, have not been previously assessed. To determine the added value of radiomic phenotypes, to ctDNA data and established clinical prognostic covariates retained from univariable modeling, we next calculated multivariable Cox regression models that incorporated number of ctDNA-detected mutations, smoking status, and ECOG performance score, both with and without radiomic phenotype.

The PFS model without phenotype yielded a c-statistic of 0.73 (95% CI 0.59–0.86); a model using radiomic phenotype alone gave a c-statistic of 0.63 (95% CI 0.49–0.77); and including radiomic phenotype in the multivariable model increased the c-statistic to 0.77 (95% CI 0.64–0.89) with an LRT *p* < 0.005, suggesting that this model had a better fit than the model without phenotype (Table 4). The pattern was similar for OS. The OS multivariable model without radiomic phenotype yielded a c-statistic of 0.8 (95% CI 0.61–0.98); the model using phenotype alone had a c-statistic of 0.62 (95% CI 0.39–0.85); and adding radiomic phenotype to the multivariable model increased the c-statistic to 0.83 (95% CI 0.67–1) with an LRT *p* = 0.08 (Table 4).

**Table 4:** Predictive ability of Cox regression models for progression-free and overall survival.

Modeling covariates	C-statistic (95% CI)	*p* versus null^1^	*p* versus model without phenotype^2^
**Progression-free survival**			
Number of mutations	0.50 (0.37–0.63)	0.97	
Smoking status	0.63 (0.47–0.78)	0.09	
ECOG performance score	0.69 (0.58–0.8)	< 0.005	
Radiomic phenotype	0.63 (0.49–0.77)	0.03	
Number of mutations, smoking status, and ECOG performance score	0.73 (0.59–0.86)	< 0.005	
Radiomic phenotype, number of mutations, smoking status, and ECOG performance score	0.77 (0.64–0.89)	< 0.005	0.01
**Overall survival**			
Number of mutations	0.55 (0.31–0.8)	0.46	
Smoking status	0.69 (0.49–0.89)	0.02	
ECOG performance score	0.71 (0.55–0.88)	0.02	
Radiomic phenotype	0.62 (0.39–0.85)	0.11	
Number of mutations, smoking status, and ECOG performance score	0.80 (0.61–0.98)	0.01	
Radiomic phenotype, number of mutations, smoking status, and ECOG performance score	0.83 (0.67–1)	< 0.005	0.08

^1^p value by likelihood ratio test versus the hypothesis that the model is no better than the null model, in which all patients are at equal risk.

^2^p value by likelihood ratio test versus the hypothesis that the model is no better than the same model without radiomic phenotype.

**Figure 6:**
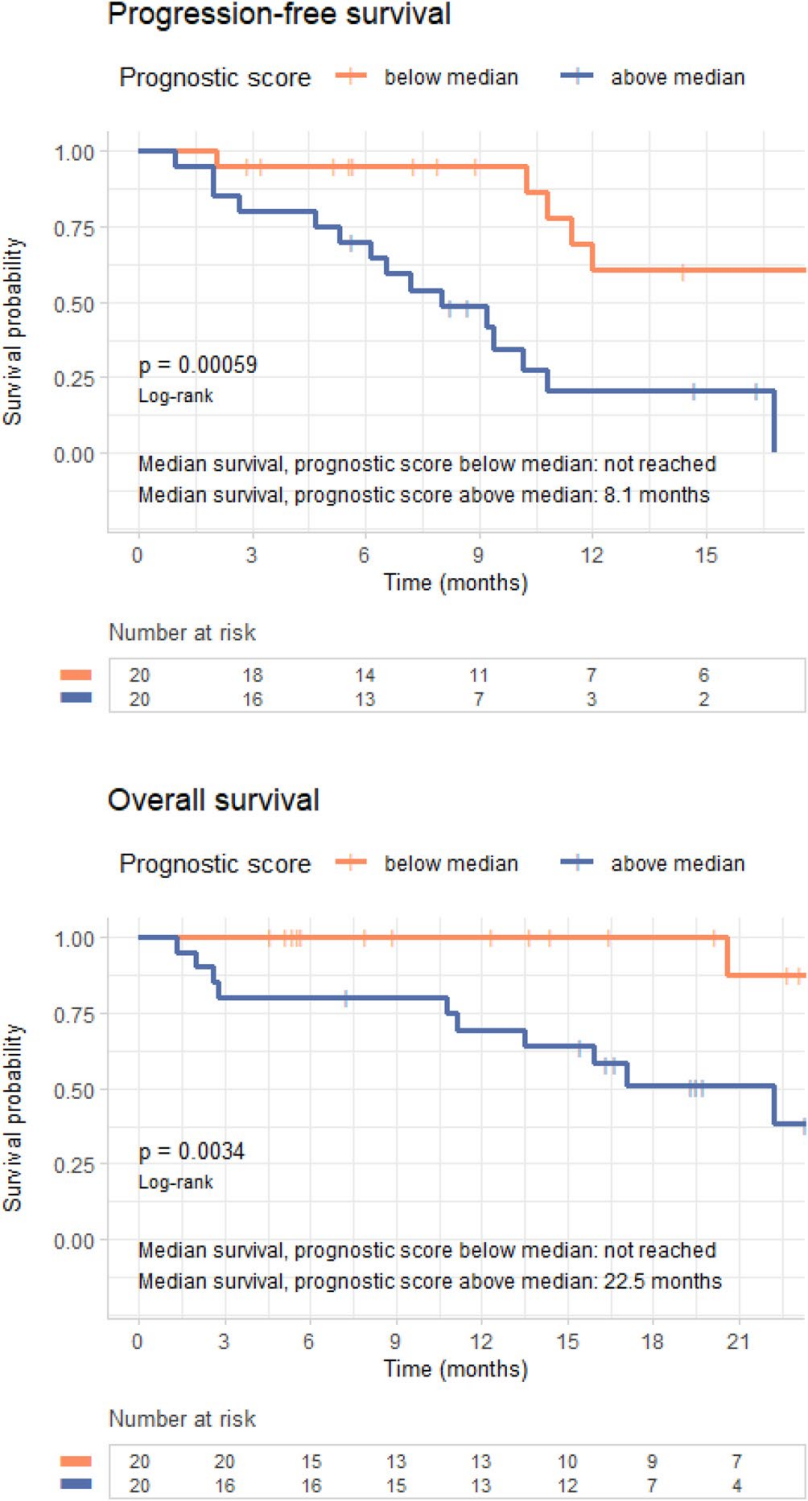
Survival analysis using multivariable model. Progression-free survival (top) and overall survival (bottom) analysis for the full multivariable model, including number of mutations, smoking status, Eastern Cooperative Oncology Group (ECOG) performance score, and radiomic phenotype.

The full multivariable model of PFS, incorporating number of mutations, smoking status, ECOG performance score, and radiomic phenotype, yielded *p* < 0.005 for separation of K–M curves for patients above versus below the median prognostic score (Fig. 6). Of the covariates in this model, only ECOG performance status (HR 5.1 (95% CI 2.0–13.3) for each increment in grade, *p* < 0.005) and phenotype (HR 3.8 (95% CI 1.3–10.7) for tumors in radiomic phenotype 2 versus 1, *p* = 0.01) had statistically significant association for HR ≠ 1 (Table 2). The full multivariable model of OS also had *p* < 0.005 for separation of the K–M curves for patients above versus below the median prognostic score (Fig. 6). Of the covariates included, only ECOG performance status (HR 4.4 (95% CI 1.2, 16.6) for each increment in grade, *p* = 0.03), had statistically significant association for HR ≠ 1. (Table 3).

## Discussion

We have used computerized tomography (CT) images to identify patient subpopulations with radiomic phenotypes that show differing responses to epidermal growth factor receptor (EGFR) tyrosine kinase inhibitors (TKIs). In particular, we combined several non-invasively gathered prognostic factors: clinical data from electronic medical records, circulating-tumor DNA (ctDNA) next-generation sequencing (NGS) ordered as standard of care, and radiomic features extracted from clinically acquired chest CT scans. A model including radiomic phenotype, number of mutations, smoking status, and ECOG performance score had better performance in predicting PFS than a model without radiomic phenotype, increasing the c-statistic from 0.73 to 0.77 (LRT *p* = 0.01). Similarly, for predicting OS, adding radiomic phenotype raised the c-statistic from 0.80 to 0.83 (LRT *p* = 0.08). Both augmented models showed statistically significant separation of K-M curves when split at their median prognostic score (*p* < 0.005 for both).

Although TKIs have dramatically changed the management of metastatic non-small cell lung cancer (NSCLC)^5,8,44^, the detection of a driver *EGFR* mutation in tumor tissue or ctDNA is necessary but insufficient for predicting response^6,12^. More than half of patients will experience initial response, but a substantial proportion will exhibit de novo or acquired resistance^4^. In addition, tumor tissue sampling can be difficult or impossible to access, especially for metastatic disease^12^. Therefore, there is an urgent need for non-invasive measures to more effectively stratify patients on to targeted therapy. Although studies suggest promising roles for both ctDNA and radiomics in complementing tissue biopsy, both have limitations when used in isolation: ctDNA sensitivity is less than ideal^13^, and radiomics are difficult to interpret in the absence of biologic correlates^20^. Finding useful radiomic signatures is also a substantial challenge, as the number of radiomic features continues to grow. In this study, we used correlation-based hierarchical clustering and principal component analysis to first mitigate feature dimensionality and then define distinct radiomic phenotypes of tumors based on the derived feature signatures.

While most previously published studies have focused on determining associations between radiomic features and *EGFR* mutation status^19,21–29,32^, which is a surrogate marker of TKI response, to the best of our knowledge our study is one of the first to evaluate the feasibility of combining radiomic features and mutation status data acquired from liquid biopsy to directly predict patient outcomes after EGFR-TKI therapy. In addition, while most prior studies have examined associations between individual radiomic features and *EGFR* mutations, our study sought to identify phenotypic signatures that represent intrinsic patterns in radiomic data. Our analysis showed a trend for association for radiomic phenotype with *EGFR* T790M mutation (*p* = 0.07), which is in line with prior studies^20,32^, although not specific to *EGFR* T790M. If further validated, radiomic analysis could provide an inexpensive, fast, and clinically feasible tool to identify patients at high risk of developing resistance mutations.

**Figure 7:**
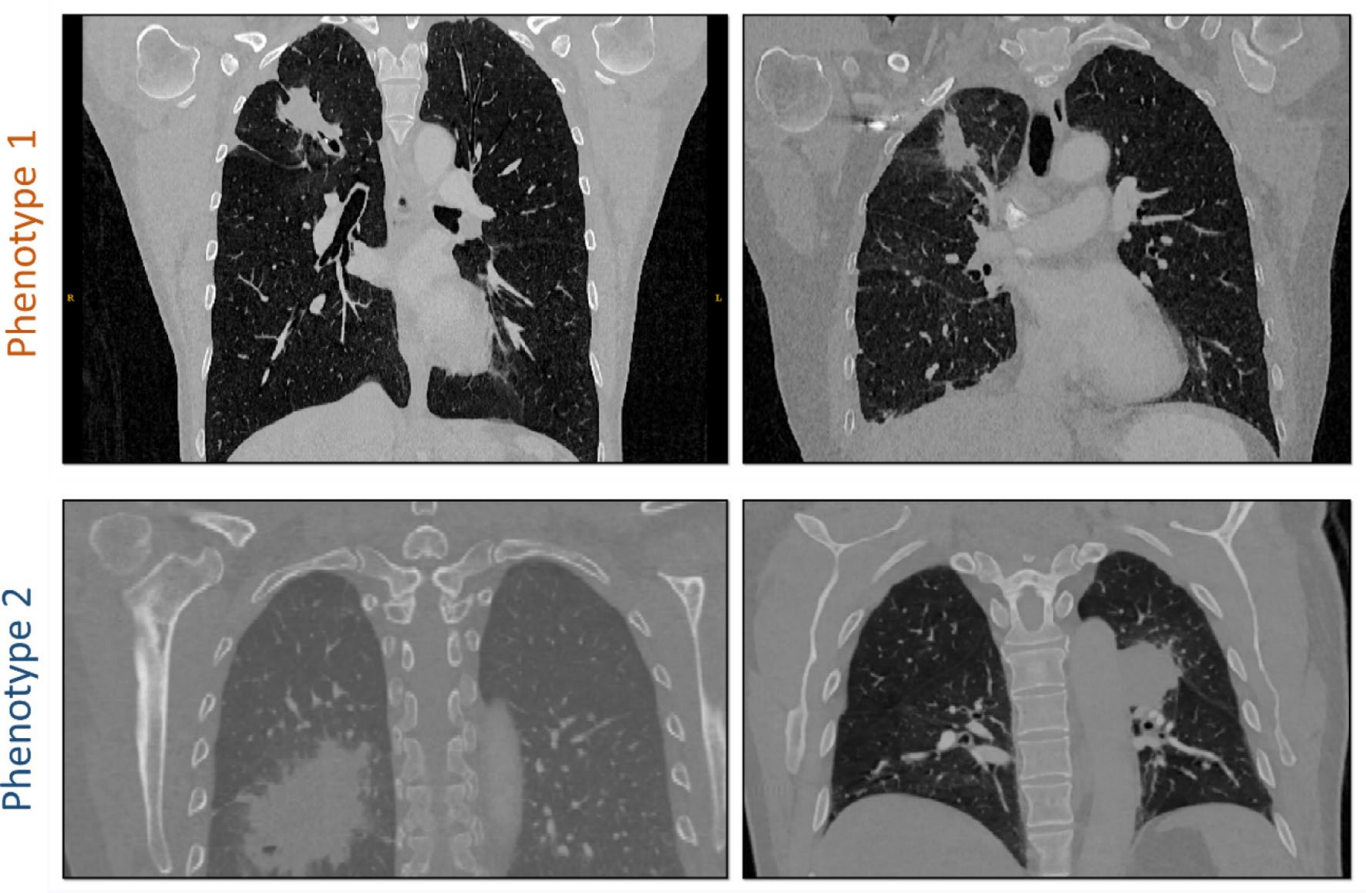
Representative tumors from the two phenotypes. Examples demonstrate the relatively smaller, elongated shape, convex borders and adjacent linear opacities for phenotype 1 versus the larger size, ground-glass, irregular, and indistinct border characteristics for phenotype 2 suggestive of potential inflammatory changes that may be related to their observed worse PFS and OS outcomes.

Our study also found a statistically significant association between phenotypes and first versus later lines of TKI therapy. Interestingly, phenotype 1 which had better PFS and OS outcomes had a higher number of second and third line therapy patients (62%), whereas phenotype 2 which had worse outcomes had a higher number of first-line patients (79%). One explanation may be that radiomic phenotypes may be a surrogate of tumor heterogeneity. Such heterogeneity has been associated with inferior response and outcomes in patients receiving EGFR TKIs^3^. When visually examining the detected phenotypes, we observed that most cancers in phenotype 1 appear to be relatively smaller, with elongated shape, convex borders and adjacent linear opacities, while cancers in phenotype 2 appear to be generally larger, and have more ground-glass, irregular, and indistinct border characteristics (Fig. 7, Supplementary Fig. S2), suggestive of potential inflammatory changes that may be related to their worse outcomes. At the same time, the characteristics of the cancers clustered in phenotype 1 may potentially also reflect the effect of prior therapy for the 13 of 17 patients receiving later line therapy.

Limitations of our study must also be noted. Our study sample is relatively small. As a proof of concept, it is important that our findings must be validated in larger future studies with independent cohorts. In addition, we used manual segmentation of tumors by only one human expert. While studies have shown that in general tumor segmentation and radiomic feature extraction could be affected by inter-rater variability^45^, recent studies suggest that such variability may not necessarily affect the robustness of all radiomic features^46^. In a preliminary evaluation, we also recently showed that despite inter-reader variation, radiomic features extracted from segmentations obtained by different human raters tend to be highly correlated and have similar predictive value^47^. Our future larger studies should seek to further evaluate the effect of reader segmentation on radiomic features, and ideally utilize fully-automated algorithms. Our study also combines radiomic features from both contrast-enhanced and non-contrast enhanced CT scans as well as from different scanners and acquisition protocols. While acknowledging that such acquisition factors may have an effect on the extracted radiomic features, our analysis showed that the use of contrast agent, spiral pitch, X-ray tube voltage and current did not appear to confound the detected phenotypes. Nevertheless, our relatively small sample size did not confer statistical power to rigorously perform stratified analysis across all possible acquisition factors to fully evaluate image acquisition effects. We are encouraged that despite the potential noise introduced by such effects we were able to detect radiomic phenotypes with statistically significant associations with outcomes and plan to further explore the effect of CT acquisition on radiomic phenotypes in our future larger studies. Finally, our study sample included a mix of patients who had received either first or later line TKI, with our models being more strongly predictive of survival for the latter group. Nevertheless, despite this heterogeneity of patients, our fully-combined multivariable model can more accurately predict survival than any one set of covariates alone.

Our study suggests that radiomic features may augment liquid biopsy and clinical prognostic factors to enhance precision oncology approaches for the management of advanced non-small cell lung cancer (NSCLC) patients. If validated, these radiomic phenotypes could be used to identify the subgroup of patients with less favorable outcomes to tyrosine kinase inhibitor (TKI) therapy who might benefit from combination therapy. Recently, for epidermal growth factor receptor (EGFR)-mutated NSCLC, the EGFR-TKI, osimertinib, has transitioned to the front-line treatment of choice based on the FLAURA trial^48,49^ and studies evaluating our radiomic phenotypes in this setting are ongoing. Future work, will include an extension of this approach to other recently approved targeted therapies, such as the use of osimertinib as a front-line EGFR inhibitor, and TKIs targeting other mutations such as EML4-ALK and ROS1 translocations. Ultimately, our work could pave the way for application in broader settings for patients suffering from advanced NSCLC as well as other solid tumors for which targeted therapies are approved.

## Supplementary Information

Supplementary Information.

## Abbreviations

NSCLC: Non-small lung cancer
EGFR: Epidermal growth factor receptor
ctDNA: Circulating-tumor DNA
K-M: Kaplan–Meier (K–M)
PFS: Progression-free survival
OS: Overall survival
LRT: Likelihood ratio test
TKI: Tyrosine kinase inhibitor
HRs: Hazard ratios
ECOG: Eastern Cooperative Oncology Group
