# Supplementary material for: Combining radiomic phenotypes of non-small cell lung cancer with liquid biopsy data may improve prediction of response to EGFR inhibitors
Source: Sci Rep. 2021 May 11;11:9984. doi: 10.1038/s41598-021-88239-y (PMC8113313; doi:10.1038/s41598-021-88239-y)
Supplement: Supplementary file 1 — Supplementary Information. [file 41598_2021_88239_MOESM1_ESM.pdf]

Combining radiomic phenotypes of non-small cell lung cancer with liquid biopsy data may improve prediction of response to *EGFR* inhibitors

Bardia Yousefi [1], Michael J. LaRiviere [2], Eric A. Cohen [1], Thomas H. Buckingham [3], Stephanie S. Yee [3], Taylor A. Black [3], Austin L. Chien [3], Peter Noël [1], Wei-Ting Hwang [4], Sharyn I. Katz [1], Charu Aggarwal[3], Jeffrey C. Thompson [5], Erica L. Carpenter [3§], Despina Kontos [1§*]

[1] Department of Radiology, University of Pennsylvania, Philadelphia, PA 19104
[2] Department of Radiation Oncology, University of Pennsylvania, Philadelphia, PA 19104
[3] Department of Medicine, Division of Hematology and Oncology, University of Pennsylvania, Philadelphia, PA 19104
[4] Department of Biostatistics, Epidemiology and Informatics, University of Pennsylvania, Philadelphia, PA 19104
[5] Department of Medicine, Section of Interventional Pulmonology, University of Pennsylvania, Philadelphia, PA 19104

[§] co-senior authors
*** Corresponding author:**

Despina Kontos
Computational Biomarker Imaging Group (CBIG)
University of Pennsylvania, Department of Radiology
Rm D702 Richards Bldg., 3700 Hamilton Walk
Philadelphia, PA 19104
Office telephone: 215-746-4064
Telephone of the laboratory: 215-746-4060
Email: Despina.Kontos@pennmedicine.upenn.edu

Supplemental Material

Table S1: Imaging acquisition parameters for CT scans

CT imaging parameters		
**Characteristic**	Siemens Healthineers	GE Medical Systems
**Scanner**	24	16
**Contrast Enhanced (CE)**		
**CE**	20	13
**Non-CE**	4	3
**Convolution Kernel**		
**Standard (soft-tissue)**	8	4
**Hard (lung)**	16	12
**Exposure (*mAs*)**	109 (43, 242)	7 (1,74)
**Focal Spots (*mm*)**		
**0.8**	4	2
**1.2**	20	14
**Tube Voltage (*kVp*)**	100 (90,140)	120
**Tube Current *(mA)***	184 (69,2383)	150 (114,649)
**Spiral Pitch Factor**	0.8 (0.6, 2.45)	1.07 (0.98,1.38)
**Unspecified cases**	-	6
**Reconstruction Diameter (*mm*)**	401 (324,500)	483 (349,483)

Table S2: Association of contrast-enhanced versus non-contrast-enhanced image type with radiomic phenotype

	Non-contrast-enhanced	Contrast-enhanced	p[1]
	***n* (percent of phenotype)**		
phenotype 1	4 (19%)	17 (81%)	1
phenotype 2	3 (15.8%)	16 (84.2%)	

1. p value by Fisher's exact test.

Table S3: Association of other CT parameters with radiomic phenotype

parameter	p[1]
Spiral pitch	0.26
Tube voltage	0.13
Tube current	0.68

1. p value by Mann-Whitney-Wilcoxon test, two-sided.

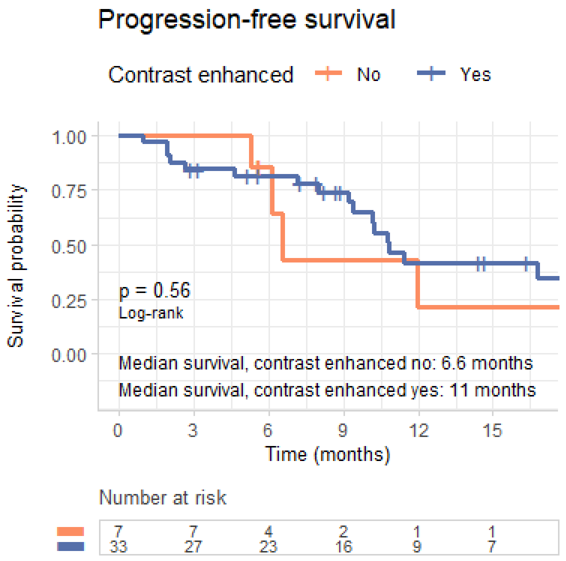

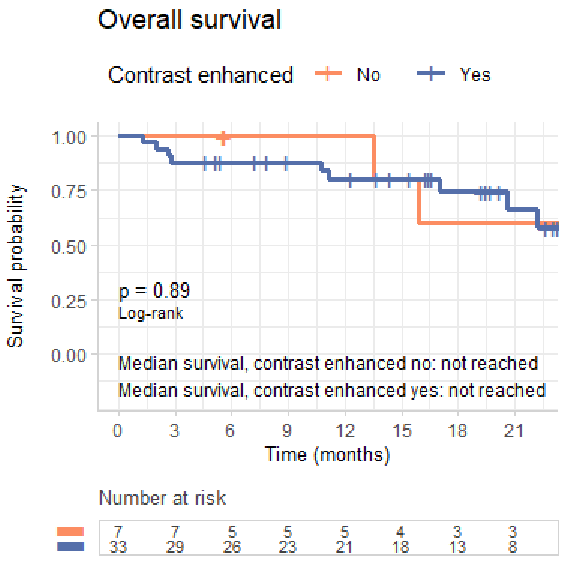

Figure S1: Association of contrast-enhanced versus non-contrast-enhanced image type with progression-free and overall survival outcomes

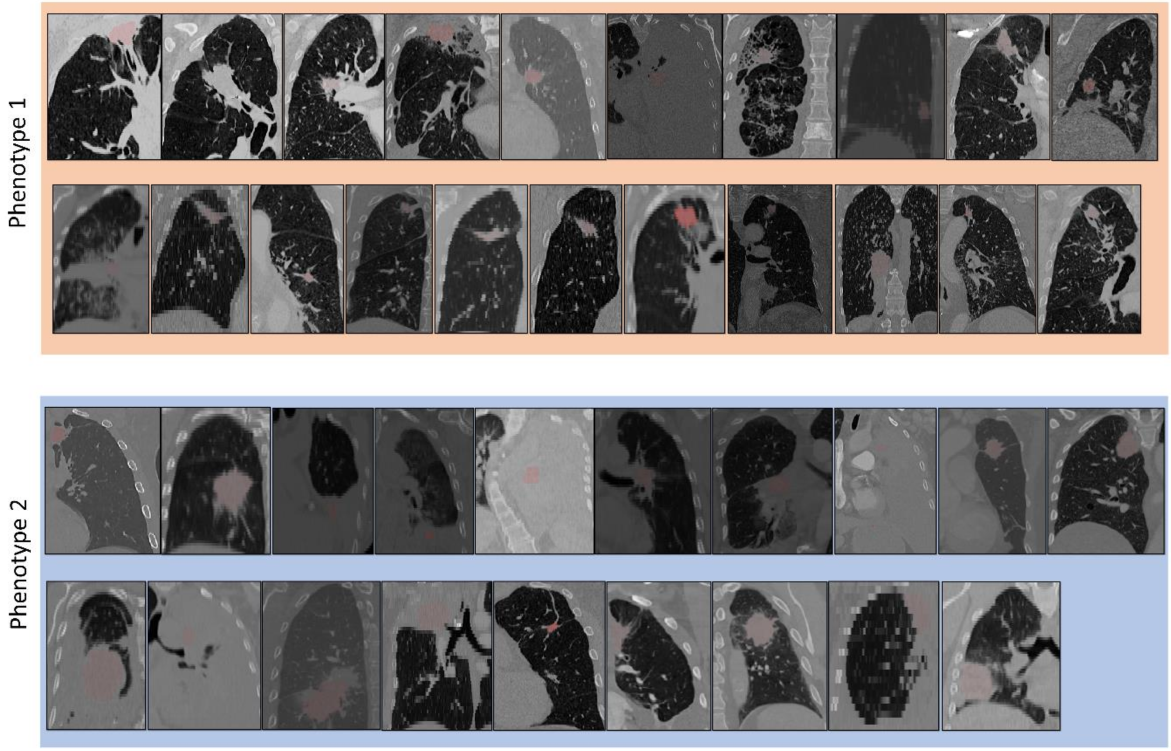

Figure S2: Tumors cluster in the two phenotypes. Visualizations of the original CT images with tumors in field of view for phenotypes 1 (n=21) and 2 (n=19). Most cancers in phenotype 1 appear to be relatively smaller, with elongated shape, convex borders and adjacent linear opacities, while cancers in phenotype 2 are generally larger and have more ground-glass, irregular, and indistinct border characteristics suggestive of potential inflammatory changes that may be related to their observed worse PFS and OS outcomes. The tumor area is highlighted by 5-10% opacity for demonstration purposes.
